# A high hematopoietic cell transplantation comorbidity Index (HCT-CI) does not impair outcomes after non-myeloablative allogeneic stem cell transplantation in acute myeloid leukemia patients 60 years or older

**DOI:** 10.1038/s41409-022-01833-0

**Published:** 2022-10-04

**Authors:** Donata Backhaus, Dominic Brauer, Rosmarie Pointner, Lara Bischof, Vladan Vucinic, Georg-Nikolaus Franke, Dietger Niederwieser, Uwe Platzbecker, Madlen Jentzsch, Sebastian Schwind

**Affiliations:** Medical Clinic and Policlinic 1, Hematology, Cellular Therapy and Hemostaseology; University of Leipzig Medical Center, Leipzig, Germany

**Keywords:** Acute myeloid leukaemia, Risk factors, Stem-cell therapies

## Abstract

For most acute myeloid leukemia (AML) patients an allogeneic hematopoietic stem cell transplantation (HSCT) offers the highest chance of cure. The introduction of less toxic non-myeloablative conditioning (NMA) regimes enabled older and/or comorbid patients to be consolidated with an allogeneic HSCT. While the hematopoietic cell transplantation comorbidity index (HCT-CI) predicted outcomes in many younger patient cohorts its impact in older AML patients receiving NMA-HSCT remains unknown. Here we analyzed 289 AML patients 60 years or older (median age 66, range 60-77 years) undergoing NMA-HSCT (2 or 3 Gray total body irradiation and 3 days of fludarabine 30 mg/m^2^). HCT-CI risk was low, intermediate, or high in 36%, 31%, and 33% of patients, respectively. Non-relapse mortality (NRM), cumulative incidence of relapse (CIR), and overall survival (OS) did not differ between HCT-CI groups. The HCT-CI also did not impact outcomes when considering the European LeukemiaNet 2017 risk at diagnosis or the measurable residual disease (MRD) status at HSCT. Notably, MRD-negative older NMA-transplanted AML patients had a beneficial OS of 49% after 5 years. Since a higher HCT-CI did not impair outcomes, age or comorbidities per se should not impede NMA-HSCT, presenting a feasible consolidation option for this group of AML patients.

## Introduction

Acute myeloid leukemia (AML) is an aggressive, clonal myeloid disorder with heterogeneous disease courses [1, 2]. Treatment options comprise chemotherapy, targeted agents, and—representing the therapy with the highest chance of cure for most patients—allogeneic hematopoietic stem cell transplantation (HSCT) [3]. Since the median age at AML diagnosis is over 65 years [4, 5], patients are often affected by additional comorbidities. Age and comorbidities may restrict the use of an allogeneic HSCT due to toxic effects of the conditioning regimens and the possibility of significant graft-versus-host disease (GvHD) leading to increased non-relapse mortality (NRM). To render older and comorbid AML patients suitable for a consolidating allogeneic HSCT, non-myeloablative (NMA) conditioning regimens with very low toxicity were developed [6, 7].

However, selecting patients for a consolidating HSCT considering relapse risks as well as the transplant associated morbidity and mortality remains a complex challenge for the treating physician team. To help inform this decision the hematopoietic cell transplantation comorbidity index (HCT-CI) score was established to predict NRM following allogeneic HSCT, replacing the more unspecific Charlson Comorbidity Index [8]. The initial cohort to evaluate the HCT-CI included younger patients with a median age of 45 years with different lymphoid, myeloid, and non-malignant hematologic diseases and conditioning regimens of varying intensity [9]. The seventeen items of the score were selected and weighted based on hazard ratios for each analyzed comorbidity. Analyses of outcomes in other hematologic diseases subjected to reduced intensity (RIC) or NMA-HSCT confirmed the usability of the HCT-CI for NRM and overall survival (OS) prediction in patients with a median age under 60 years [10–12]. On the other hand some additional HSCT studies demonstrated only a limited ability for NRM and OS prediction of the score [13–20], and none of the studies analyzed older AML patients receiving low toxicity NMA-HSCT. Here we analyzed the impact of the commonly used HCT-CI score on NRM and OS, also in the context of newer prognostic factors, as the current European LeukemiaNet 2017 (ELN2017) risk stratification and the measurable residual disease (MRD) status at HSCT in older AML patients following NMA-HSCT.

## Subjects and methods

### Patients and treatment

We retrospectively analyzed 289 AML patients 60 years or older (median age at transplantation 66, range 60–76) subjected to NMA-HSCT between 1999 and 2018 at the University Hospital Leipzig and for whom full comorbidity assessment according to the HCT-CI score were available. CIR and NRM per timepoint of transplantation are displayed in Supplementary Fig. S1. The NMA conditioning regimen consisted of fludarabine 30 mg/m^2^ for three consecutive days and 2 (*n* = 278) or 3 Gray (*n* = 6) total body irradiation (TBI), 5 patients received 2 Gray TBI alone. GvHD prophylaxis contained cyclosporine and mycophenolate mofetil (for details see Supplementary Information). Reasons to assign patients to NMA conditioning, according to institutional guidelines at that time, were age older than 55 years for related HSCT (*n* = 50) or older than 50 years for unrelated HSCT (*n* = 237) or performing a second allogeneic HSCT after HSCT for preceding myelodysplastic syndrome (MDS, *n* = 2). Median follow up after HSCT was 3.8 years. Further patients’ characteristics are shown in Table 1.

**Table 1:** Clinical, genetic, and HSCT-related characteristics of NMA-HSCT treated patients according to the HCT-CI risk score (*n* = 289).

	HCT-CI 0 points (*n* = 104)	HCT-CI 1/2 points (*n* = 90)	HCT-CI ≥ 3 points (*n* = 95)	P* (0 *vs* 1/2)	P* (1/2 *vs* 3)	P* (0 *vs* 3)
Clinical characteristics						
Age at diagnosis, years						
Median	63	66	66	0.28	0.41	0.06
Range	54–74	58–76	60–76			
Age at transplant, years						
Median	64	66	66	0.37	0.57	0.12
Range	60–75	60–76	60–76			
Sex, *n* (%)						
Male	54 (51.9)	59 (65.6)	45 (47.4)	0.06	0.02	0.57
Female	50 (48.1)	31 (34.4)	50 (52.6)			
Disease origin, *n* (%)						
Secondary AML	41 (39.4)	35 (38.9)	48 (50.5)	0.99	0.14	0.12
De novo AML	63 (60.6)	55 (61.1)	47 (49.5)			
Hemoglobin at diagnosis, g/dl						
Median	8.9	9.0	8.6	0.72	0.25	0.45
Range	4.3–13.4	4.5–12.7	5.3–15.3			
Platelet count at diagnosis, x 10^9^/L						
Median	67	58	72	0.70	0.10	0.26
Range	3–950	2–192	9–501			
WBC count at diagnosis, x 10^9^/L						
Median	4.5	8.5	3.2	0.12	0.01	0.42
Range	0.8–366	0.6–385	0.7–160			
Blood blasts at diagnosis, %						
Median	20	25	10	0.67	0.05	0.07
Range	0–97	0–97	0–98			
BM blasts at diagnosis, %						
Median	45	50	54	0.22	0.89	0.23
Range	3–93	18–95	16–95			
BM CD34 + /CD38- cells at diagnosis, %				0.30	0.76	0.19
Median	0.5	0.5	1			
Range	0–89	0–63	0–39			
Genetic characteristics						
ELN2017 genetic group, *n* (%)				0.70	0.14	0.27
Favorable	18 (17.3)	13 (14.4)	15 (16.7)			
Intermediate	18 (17.3)	15 (16.7)	27 (28.4)			
Adverse	32 (30.7)	33 (36.7)	26 (27.4)			
Unknown	36 (34.6)	29 (32.2)	27 (28.4)			
Complex karyotype, *n* (%)						
Absent	78 (75)	72 (80.0)	70 (73.7)	0.62	0.24	0.51
Present	12 (11.5)	8 (8.9)	15 (15.8)			
Unknown	14 (13.5)	10 (11.1)	10 (10.5)			
Core Binding Factor AML, *n* (%)						
Absent	87 (83.7)	80 (88.9)	88 (92.6)	0.61	0.61	0.99
Present	1 (1.0)	2 (2.2)	1 (1.1)			
Unknown	16 (15.4)	8 (8.9)	6 (6.3)			
*NPM1* at diagnosis, *n* (%)						
Wild-type	54 (51.9)	46 (51.1)	56 (58.9)	0.99	0.99	0.99
Mutated	17 (16.3)	15 (16.7)	18 (18.9)			
Unknown	33 (31.7)	29 (32.2)	21 (22.1)			
*FLT3*-ITD at diagnosis, *n* (%)						
Absent	61 (58.7)	44 (48.9)	59 (62.1)	0.04	0.16	0.50
Present	10 (9.6)	19 (21.1)	14 (14.7)			
Unknown	33 (31.7)	27 (30.0)	22 (23.2)			
*CEBPA* at diagnosis, *n* (%)						
Wild-type	56 (53.8)	44 (48.9)	57 (60.0)	0.82	0.60	0.35
Mutated	13 (12.5)	9 (10.0)	8 (8.4)			
Unknown	35 (33.7)	37 (41.1)	30 (31.6)			
*FLT3*-TKD at diagnosis, *n* (%)						
Absent	60 (57.7)	55 (61.1)	65 (68.4)	0.34	0.99	0.21
Present	7 (6.7)	3 (3.3)	3 (3.2)			
Unknown	37 (35.6)	32 (35.6)	27 (28.4)			
*RUNX1* at diagnosis, *n* (%)						
Wild-type	31 (29.8)	18 (20.0)	23 (100.0)	0.99	0.10	0.07
Mutated	6 (5.8)	3 (3.0)	0 (0.0)			
Unknown	67 (64.4)	69 (76.7)	72 (75.8)			
*ASXL1* at diagnosis, *n* (%)						
Wild-type	33 (31.7)	18 (20.0)	20 (21.1)	0.70	0.99	0.99
Mutated	4 (3.8)	3 (3.0)	3 (3.2)			
Unknown	67 (64.4)	69 (76.7)	72 (75.8)			
*TP53* at diagnosis, *n* (%)						
Wild-type	32 (30.8)	18 (20.0)	20 (21.1)	0.99	0.99	0.99
Mutated	5 (4.8)	3 (3.0)	3 (3.2)			
Unknown	67 (64.4)	69 (76.7)	72 (75.8)			
*IDH1* at diagnosis, *n* (%)						
Wild-type	57 (54.8)	46 (51)	37 (38.9)	0.19	0.08	0.59
Mutated	8 (7.7)	2 (2.2)	7 (7.4)			
Unknown	39 (37.5)	42 (46.7)	51 (53.7)			
*IDH2* at diagnosis, *n* (%)						
Wild-type	54 (51.9)	42 (51.1)	36 (37.9)	0.80	0.43	0.62
Mutated	11 (10.6)	7 (2.2)	10 (10.5)			
Unknown	39 (37.5)	42 (46.7)	49 (51.6)			
HSCT-related characteristics						
Disease status at HSCT, *n* (%)						
CR1	63 (61)	51 (57)	58 (43)	0.66	0.55	0.99
CR2	16 (15)	18 (20)	12 (13)	0.45	0.23	0.68
CR3	1 (1)	0 (0)	0 (0)	0.99	0.99	0.99
CRi	14 (13)	10 (11)	18 (19)	0.66	0.15	0.34
Worse	10 (10)	11 (12)	7 (7)	0.65	0.33	0.62
Chemotherapy cycles prior to HSCT, *n* (%)						
1	26 (35)	25 (28)	24 (25)			
2	59 (57)	55 (61)	47 (49)	0.41	0.04	0.44
≥3	19 (18)	10 (11)	24 (25)			
Donor match, *n* (%)						
HLA antigen matched related	19 (18)	16 (18)	15 (16)			
HLA antigen matched unrelated	54 (52)	54 (60)	59 (62)	0.44	0.96	0.33
HLA antigen mismatched	31 (30)	20 (22)	21 (22)			
Donor sex, *n* (%)						
All others	83 (80)	80 (90)	86 (91)	0.07	0.80	0.03
Female into male	21 (20)	9 (10)	8 (9)			
Regeneration after HSCT						
Platelets				0.53	0.79	0.32
Median day (range)	12 (0–28)	11 (0–21)	11 (0–27)			
WBCs				0.95	0.63	0.54
Median day (range)	13 (0–31)	13 (0–23)	13 (0–25)			

*AML* acute myeloid leukemia, *ASXL1* additional Sex Combs-Like 1 gene, *BM* bone marrow, *CEBPA* CCAAT/enhancer-binding protein alpha gene, *CR* complete remission, *CRi* complete remission with incomplete peripheral hematologic regeneration, *ELN2017* European leukemia net classification 2017, *FLT3-ITD* internal tandem duplication of fms like tyrosine kinase 3 gene, *FLT3-TKD* tyrosine kinase domain of fms like tyrosine kinase 3 gene, *HLA* human leukocyte antigen, *HSCT* hematopoietic stem cell transplantation, *IDH* isocitrate dehydrogenase gene, *NMA* non-myeloablative, *NPM1* nucleophosmin 1 gene, *RUNX1* runt-related transcription factor 1 gene, *TP53* tumor protein P53, *WBC* white blood cell count.

*P values are given for patients with data available.

Written informed consent for participation in these studies was obtained in accordance with the Declaration of Helsinki. Data analyses were approved by the institutional review board of the University Hospital Leipzig.

### Cytogenetics, molecular analyses, and immunophenotype

Cytogenetic analyses were performed using standard banding techniques. In cases where no metaphases could be obtained, fluorescence in situ hybridization (FISH) was used to screen for recurrent cytogenetic aberrations. Mutations in the genes *CEBPA*, *NPM1*, *FLT3*-ITD, *FLT3*-TKD, and *ASXL1*, as well as a next generation sequencing panel comprising 54 recurrently mutated genes in myeloid neoplasm were performed as previously described [21]. AML disease risk was assessed according to ELN2017 classification [1].

### Measurable residual disease evaluation prior to HSCT

The MRD status up to 28 days prior to HSCT was assessed in patients transplanted in CR or CR with incomplete peripheral hematologic recovery (CRi) who had adequate material available (*n* = 190). MRD was based on *NPM1* mutations [22], and *BAALC/ABL1* [23], *MN1/ABL1* [24], and *WT1/ABL1* [25] expression levels using the previously published cut-offs. Patients with at least one positive MRD test were regarded as MRD-positive [26].

### Calculation of the HCT-CI risk score

All patients were screened for relevant comorbidities up to 28 days prior to date of HSCT by anamnesis and at least by the following examinations: physical examination with neurological status, laboratory assessment (hemogram, electrolytes, glucose, liver function, renal function, inflammatory parameters, hemostasis), paranasal sinus, thoracic, and abdominal computed tomography, abdominal sonography, echocardiography, pulmonary function testing.

The HCT-CI score was calculated using the available worksheet from the Center for International Blood and Marrow Transplant Research (CIBMTR) and Medical College of Wisconsin in accordance with the well-defined comorbidities from the guidelines made by Sorror [27, 28]. Patients were grouped in three risk groups as previously published [9]. Prevalence of the comorbidities included in the HCT-CI score are shown in Table 2. For details of tumor prevalence, see Supplementary Information (Table S1 and Fig. S2).

**Table 2:** Prevalence of the comorbidities included in the HCT-CI score as defined in the worksheet from the CIBMTR (*n* = 289).

Parameter	Score value	*n* (%)
Arrhythmia	1	37 (13)
Cardiovascular	1	57 (20)
Inflammatory bowel disease	1	1 (<1)
Diabetes	1	37 (13)
Cerebrovascular	1	16 (6)
Depression/Anxiety	1	3 (1)
Hepatic		
Mild	1	1 (<1)
Moderate/severe	3	0 (0)
Obesity	1	8 (3)
Infection	1	90 (31)
Rheumatologic	2	4 (1)
Peptic ulcer	2	4 (1)
Renal	2	0 (0)
Pulmonary		
Moderate	2	63 (22)
Severe	3	93 (32)
Heart valve disease	3	5 (2)
Prior solid malignancy	3	68 (24)

### Endpoints and statistical analysis

All statistical analyses were performed using the R statistical software platform (version 4.3.2) [29]. CR was defined as described in the Supplementary Information. OS was calculated from HSCT to death from any cause using the Kaplan-Meier method and groups were compared using the log-rank test [30]. CIR was calculated from HSCT to relapse considering the competing risk NRM using the Fine and Gray model [31]. Associations with baseline clinical, demographic, and molecular features were compared using the Kruskal-Wallis test and Fisher’s exact test for continuous and categorical variables, respectively.

## Results

### Patients’ characteristics within the three HCT-CI risk groups

Age, sex, blood counts, ELN2017 genetic risk groups at diagnosis, the remission and MRD status prior to HSCT, as well as donor and recipient match were well balanced between the three HCT-CI groups. Further details are shown in Table 1.

### Incidences of comorbidities

Compared to the initial evaluation by Sorror et al. [9], most included comorbidities were significantly more frequent in our patient set: infection (31% vs. 4%, with *P* < 0.01), severe pulmonary function disturbances (32% vs. 9%, *P* < 0.01), cardiovascular disease (20% vs. 5%, *P* < 0.01), arrhythmia (13% vs. 5%, *P* < 0.01), cerebrovascular disease (6% *vs.* <1%, *P* < 0.01), diabetes (13% *vs*. 3%, *P* < 0.01) and prior solid tumor (24% *vs*. 2%, *P* < 0.01). However, we did not find as many psychiatric disorders and mild hepatic impairments (1% *vs.* 9%, *P* < 0.01, and 0.3% vs. 16%, *P* < 0.01).

### Outcomes of the whole patient cohort

97% of the patients (*n* = 281) engrafted. 5-year-OS of the whole cohort was 41% (95% Confidence interval [CI] 36-49%, Supplementary Fig. S3A).

**Fig. 1:**
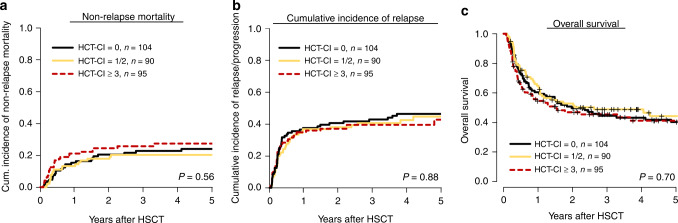
Outcome according to the HCT-CI. Non-Relapse Mortality (**a**), Cumulative Incidence of Relapse (**b**), and Overall Survival (**c**) after non-myeloablative hematopoietic stemm cell transplantation (NMA-HSCT) according to the three hematopoietic cell transplantation comorbidity index (HCT-CI) risk groups (*n* = 289).

Overall, 36% of patients had a low risk HCT-CI score (0 points, *n* = 104), 31% an intermediate (1–2 points, 60 patients had 1 point and 30 patients had two points), and 33% a high risk (≥3 points, 63 patients had 3 points, 23 patients had 4 points, 5 patients had 5 points, 3 patients had 6 points and 1 patient had 7 points). Evaluating the HCT-CI as continuous variable the score was not significantly associated with NRM (*P* = 0.63) or OS (*P* = 0.29). Additionally, we observed an area under the curve (AUC) of 0.50 for predicting NRM, and of 0.50 for predicting OS (Supplementary Fig. S4). CIR (*P* = 0.88), NRM (*P* = 0.56) and OS (*P* = 0.70) did not differ significantly between the three HCT-CI risk groups (0 vs 1/2 vs ≥ 3 points, Fig. 1a–c). After 5 years, CIR was 46%, 45%, and 43%, in the HCT-CI low, intermediate, and high risk group, respectively. NRM after 5 years was 24%, 20%, and 27%, respectively, and OS was 40%, 44%, and 41%, respectively. For incidences of limited and extensive chronic graft-versus-host disease (GvHD), see Supplementary Table S2.

### Causes of death

Overall, 56% of AML patients deceased during the follow-up time. There was no difference regarding the causes of death between the three HCT-CI risk groups (*P* = 0.39, *P* = 0.56, *P* = 0.49 respectively). In patients with low, intermediate, or high risk according to the HCT-CI risk, 53%, 59%, and 55% died after relapse, 17%, 20%, and 27% died due to GvHD without suffering relapse, 22%, 10%, and 14% died from infection without suffering relapse or GvHD, and 7%, 10%, and 4% died due to other reasons (low risk HCT-CI: two patients from secondary malignancy and two patients from cardiovascular events, intermediate risk HCT-CI: one patient from secondary malignancy and four patients from cardiovascular events, and high risk HCT-CI: one patient from secondary malignancy and one patient from cardiovascular events). For details see Supplementary Table S1 and S2).

### Subgroup analysis within the ELN2017 and MRD risk groups

**Fig. 2:**
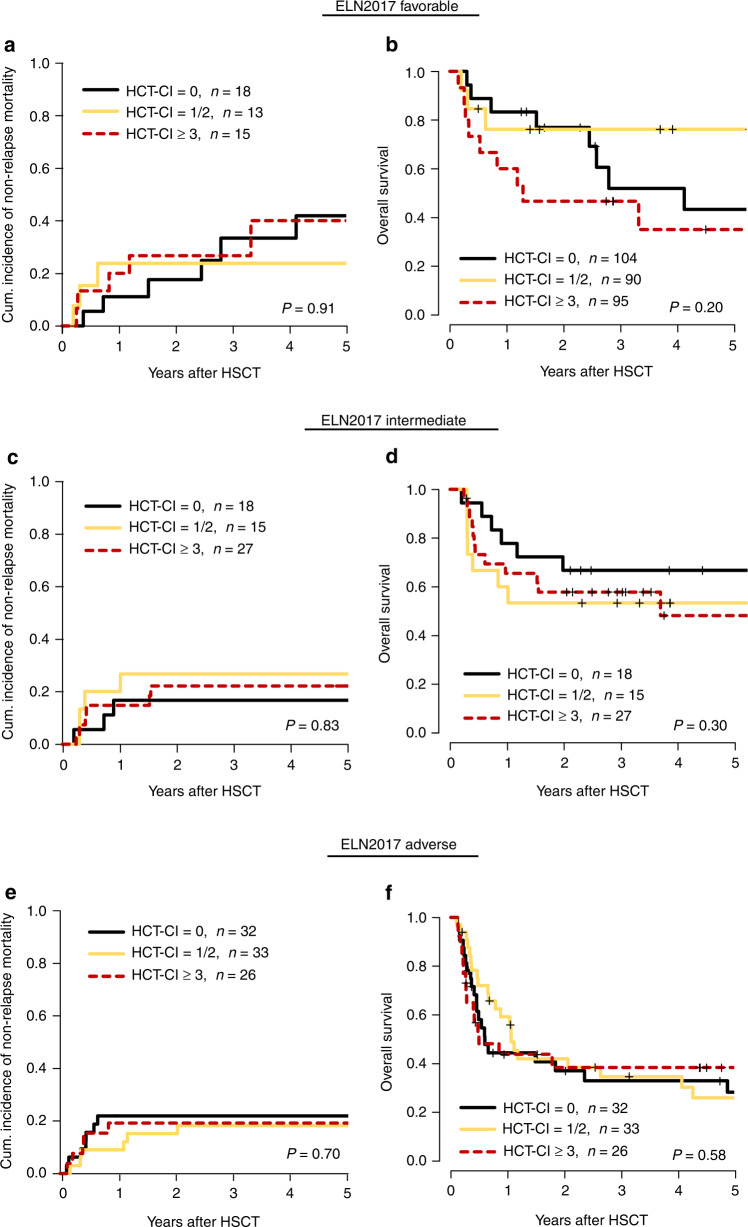
Outcome according to the HCT-CI in ELN2017 risk groups. Subanalyses of the impact of the hematopoietic cell transplantation comorbidity index (HCT-CI) score on outcome in ELN2017 classification risk groups (favorable *n* = 46, intermediate *n* = 60, adverse *n* = 91). Cumulative incidence of non-relapse mortality (CIR) (**a**) and overall survival (OS) (**b**) in ELN2017 favorable risk group, CIR (**c**) and OS (**d**) in ELN2017 intermediate risk group, CIR (**e**) and OS (**f**) in ELN2017 adverse risk group.

**Fig. 3:**
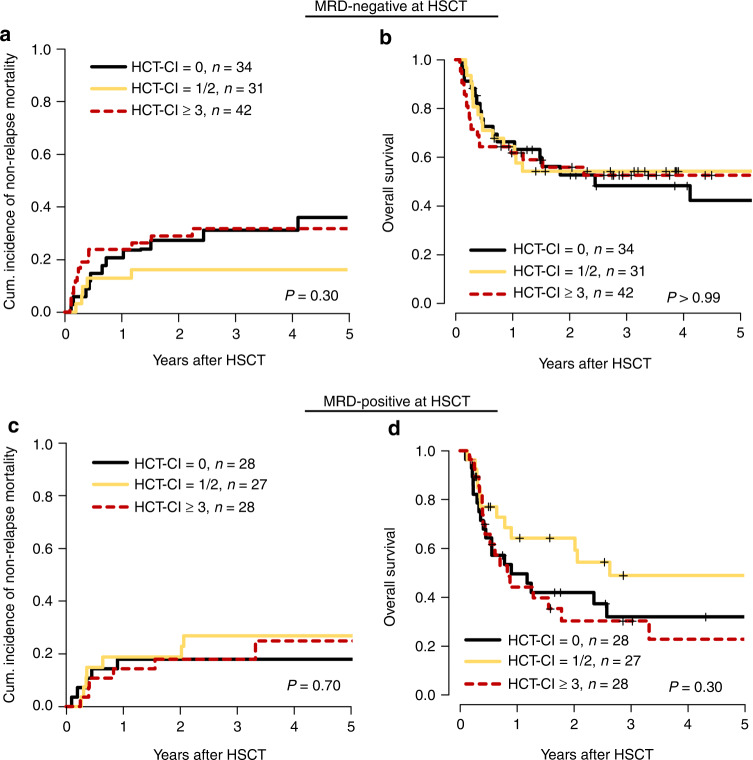
Outcome according to the HCT-CI dependent on the MRD status. Subanalyses of the impact of the hematopoietic cell transplantation comorbidity index (HCT-CI) score on outcome depending on the measurable residual disease (MRD) status at non-myeloablative hematopoietic stemm cell transplantation (NMA-HSCT) (MRD positive *n* = 83, MRD negative *n* = 107). Cumulative incidence of non-relapse mortality (CIR) (**a**) and overall survival (OS) (**b**) in MRD-negative patients at HSCT. CIR (**c**) and OS (**d**) in MRD-positive patients at HSCT.

The HCT-CI also did not significantly influence outcomes in separate analyses within the three ELN2017 risk groups (ELN20117 favorable: NRM, *P* = 0.91 and OS, *P* = 0.20, ELN2017 intermediate: NRM, *P* = 0.49 and OS, *P* = 0.30, ELN2017 adverse, NRM, *P* = 0.58 and OS, *P* = 0.70, Fig. 2a–f), or in separate analyses for patients with secondary AML (NRM, *P* = 0.16, OS, *P* = 0.10, Supplementary Fig. S5A,B), and *de novo* AML (NRM, *P* = 0.94, OS, *P* = 0.80, Supplementary Fig. S5C, D). Similarly, the HCT-CI did not impact outcomes in patients with a positive (NRM, *P* = 0.30 and OS, *P* = 0.99) or negative MRD status (NRM, *P* = 0.70 and OS, *P* = 0.30) at HSCT (Fig. 3a–d).

Noteworthy, in the subgroup of patients achieving a negative MRD status at NMA-HSCT, the median OS was beneficial and reached 49% at 5 years after HSCT (95% CI 39–61%), and was similar with respect to the assigned HCT-CI risk score (OS at 5 years: HCT-CI low risk 42%, intermediate risk 53%, and high risk 53%, Supplementary Fig. S3B).

## Discussion

For most AML patients a consolidating HSCT presents a curative therapy option. Today conditioning regimens with low toxicity enable also older and/or comorbid individuals to receive an allogeneic HSCT [32]. However, the question whether an AML patient is eligible for an allogeneic HSCT remains challenging. To aid informing such decisions, clinicians rely on tools such as the HCT-CI score, which today is widely adopted.

Here we evaluated the clinical relevance of the HCT-CI score in older (≥60 years) AML patients consolidated by NMA-HSCT. Overall outcomes of our older and comorbid AML patient cohort stand in line with previously published studies reported on similar cohorts [15, 33–35]. As expected, a significant portion of our patient cohort harbored comorbidities, which—apart from psychiatric disorders and mild hepatic impairment—were more frequent in our patient set compared to the initial evaluation by Sorror et al. [9].

However, prevalences of comorbidities in our study are similar to those shown by others [35, 36], including the relatively high prevalence of moderate to severe pulmonary function which was also reported by Barba et al. in a study of patients who received RIC-HSCT [37].

Although the prevalence of comorbidities was high, in our older AML patient set NRM following NMA-HSCT was relatively low. We did not find significant differences in CIR, NRM, or OS according to the three HCT-CI risk groups, which we relate to the low toxic conditioning regimen as compared to more intensive RIC or myeloablative (MA) procedures. Noteworthy, in these patients the HCT-CI also did not modify the outcome impact of the ELN2017 risk stratification at diagnosis, *de novo* and secondary AML patients, or the MRD status at transplantation. These results were also seen when we regraded the HCT-CI score as a continuous variable (Supplementary Table S3; and Supplementary Figs S6–S8).

In line with these observations Veeraputhiran et al. showed according to the HCT-CI score no different NRM one year after NMA-HSCT (conditioning with total lymphoid irradiation and antithymocyte globulin) of patients with AML, MDS and non-Hodgkin lymphoma (NHL) [35]. Furthermore, McClune et al. reported lower NRM in NMA-HSCT compared to RIC-HSCT (*P* = 0.05) in 1080 AML and MDS patients, but the study did not report comorbidity data [38].

In contrast, a prospective study of Sorror et al. found significant different OS and NRM in the HCT-CI risk groups for 372 older patients (age 60–75) transplanted after NMA-conditioning. However, the exclusion of patients with low cardiac ejection fraction and low pulmonary diffusion capacity, the high use of related donors and the inclusion of different hematologic diseases render a comparison with our results difficult [39]. Muffly et al. showed a significant higher hazard for death in patients with hematologic malignancies older 70 years with a HCT-CI score of 3 or more points. This effect was especially seen in patients with advanced or active disease at HSCT after RIC or NMA regimens [32].

Noteworthy, some studies also described no significant different NRM or OS between HCT-CI risk groups in patients conditioned with more intensive MA or RIC regimens [15–17, 40, 41]. However, of these only Castagna et al. analyzed patients with a median age older than 60 years. Given these variable results, the ability of the HCT-CI to predict clinically meaningful NRM or OS differences seems to be modified by the underlying disease, age at HSCT and the applied conditioning intensity.

In our cohort of 289 NMA-conditioned older AML patients the HCT-CI score did not significantly predict transplant outcomes, also irrespective of the disease aggressiveness or origin. Thus, alternative factors to predict NRM in this situation may be more clinically meaningful. As an example, Muffly et al. applied geriatric assessment to better describe comorbid conditions in an analysis that included MA and NMA conditioning regimens. In this evaluation limitations in instrumental activities of daily living (IADL), slow walk speed and—as indicator for reduced mental health—low “Short Form-36 health-related quality of life” questionnaire (SF-36-MCS), were significantly associated with higher NRM [42]. Moreover, recently we and others could demonstrate that some factors not charted by the HCT-CI score, including the red cell distribution width (RDW) at AML diagnosis [43], and weight loss during AML chemotherapy before HSCT, are relevant for OS following HSCT, which was also seen in the here presented patient set (Supplementary Fig. S9) [44, 45]. With the latter being modifiable, the control of the nutritional status from diagnosis to HSCT is an important element to lower NRM in AML patients undergoing allogeneic HSCT. Furthermore, with respect to outcomes following HSCT the improvement of quality of life by reducing the rates of extended chronic GvHD e.g., by modifying immunosuppressive therapy combinations or new treatment options of opportunistic viral infections may benefit NRM [46–48].

In conclusion, this study is the first to focus on the impact of the commonly assessed HCT-CI risk score on outcomes of older AML patients after NMA-HSCT. The HCT-CI score did not significantly influence outcomes of older AML patients following NMA-HSCT in general or in any of the analyzed subgroups. Therefore, the HCT-CI score may not be the best tool to inform decisions towards an allogeneic HSCT in this group of AML patients. However, due to restrictions in patient numbers we could not analyze patients with very high HCT-CI scores separately, which may have a more severe impact on outcomes. Additional clinical or laboratory parameters may improve NRM prediction and should be explored for this purpose. Further limitations of our study are the retrospective data and analysis of patients who responded to chemotherapy prior HSCT, surviving pretreatment. Independent of comorbidities, older AML patients achieving a negative MRD status at NMA-HSCT had a median OS of 49% at 5 years. Thus, age or comorbidities (with the exception of very high HCT-CI scores) per se should not restrict NMA-HSCT, which represents a feasible consolidation option for this group of AML patients.

## Supplementary information

Supplemental material

## Data Availability

For data sharing/original data please contact: Sebastian.Schwind@medizin.uni-leipzig.de

## References

[1] Döhner H, Estey E, Grimwade D, Amadori S, Appelbaum FR, Büchner T (2017). Diagnosis and management of AML in adults: 2017 ELN recommendations from an international expert panel. Blood.

[2] Arber DA, Orazi A, Hasserjian R, Thiele J, Borowitz MJ, Le Beau MM (2016). The 2016 revision to the World Health Organization classification of myeloid neoplasms and acute leukemia. Blood.

[3] DiNardo CD, Wei AH (2020). How I treat acute myeloid leukemia in the era of new drugs. Blood.

[4] Shallis RM, Wang R, Davidoff A, Ma X, Zeidan AM (2019). Epidemiology of acute myeloid leukemia: Recent progress and enduring challenges. Blood Rev.

[5] Juliusson G, Antunovic P, Derolf A, Lehmann S, Möllgård L, Stockelberg D (2009). Age and acute myeloid leukemia: Real world data on decision to treat and outcomes from the Swedish Acute Leukemia Registry. Blood.

[6] Grigg A, Seymour JF, Roberts A, Szer J (1999). Mini-allografts’ for haematological malignancies: an alternative to conventional myeloablative marrow transplantation. Aust N. Z J Med.

[7] Niederwieser D, Maris M, Shizuru JA, Petersdorf E, Hegenbart U, Sandmaier BM (2003). Low-dose total body irradiation (TBI) and fludarabine followed by hematopoietic cell transplantation (HCT) from HLA-matched or mismatched unrelated donors and postgrafting immunosuppression with cyclosporine and mycophenolate mofetil (MMF) can induce durable complete chimerism and sustained remissions in patients with hematological diseases. Blood.

[8] Charlson ME, Pompei P, Ales KE, MacKenzie CR (1987). A New Method of Classifying Prognostic Comorbidity in Longitudinal Studies: Development and Validation. J Chronic Dis.

[9] Sorror ML, Maris MB, Storb R, Baron F, Sandmaier BM, Maloney DG (2005). Hematopoietic cell transplantation (HCT)– specific comorbidity index: a new tool for risk assessment before allogeneic HCT. Blood.

[10] Pollack SM, Steinberg SM, Odom J, Dean RM, Fowler DH, Bishop MR (2009). Assessment of the Hematopoietic Cell Transplantation Comorbidity Index in Non-Hodgkin Lymphoma Patients Receiving Reduced-Intensity Allogeneic Hematopoietic Stem Cell Transplantation. Biol Blood Marrow Transpl.

[11] Kerbauy DMB, Chyou F, Gooley T, Sorror ML, Scott B, Pagel JM (2005). Allogeneic hematopoietic cell transplantation for chronic myelomonocytic leukemia. Biol Blood Marrow Transpl.

[12] Lim ZY, Ingram W, Brand R, Ho A, Kenyon M, Devereux S (2010). Impact of pretransplant comorbidities on alemtuzumab-based reduced-intensity conditioning allogeneic hematopoietic SCT for patients with high-risk myelodysplastic syndrome and AML. Bone Marrow Transpl.

[13] Farina L, Bruno B, Patriarca F, Spina F, Sorasio R, Morelli M (2009). The hematopoietic cell transplantation comorbidity index (HCT-CI) predicts clinical outcomes in lymphoma and myeloma patients after reduced-intensity or non-myeloablative allogeneic stem cell transplantation. Leukemia.

[14] Barba P, Ratan R, Cho C, Ceberio I, Hilden P, Devlin SM (2017). Hematopoietic Cell Transplantation Comorbidity Index Predicts Outcomes in Patients with Acute Myeloid Leukemia and Myelodysplastic Syndromes Receiving CD34+ Selected Grafts for Allogeneic Hematopoietic Cell Transplantation. Biol Blood Marrow Transpl.

[15] Birninger N, Bornhäuser M, Schaich M, Ehninger G, Schetelig J (2011). The hematopoietic cell transplantation-specific comorbidity index fails to predict outcomes in high-risk AML patients undergoing allogeneic transplantation-investigation of potential limitations of the index. Biol Blood Marrow Transpl.

[16] Nakaya A, Mori T, Tanaka M, Tomita N, Nakaseko C, Yano S (2014). Does the hematopoietic cell transplantation specific comorbidity index (HCT-CI) predict transplantation outcomes? A Prospective multicenter validation study of the kanto study group for cell therapy. Biol Blood Marrow Transpl.

[17] Guilfoyle R, Demers A, Bredeson C, Richardson E, Rubinger M, Szwajcer D (2009). Performance status, but not the Hematopoietic Cell Transplantation Comorbidity Index (HCT-CI), predicts mortality at a Canadian transplant center. Bone Marrow Transpl.

[18] Yanada M, Konuma T, Mizuno S, Saburi M, Shinohara A (2021). Predicting non-relapse mortality following allogeneic hematopoietic cell transplantation during first remission of acute myeloid leukemia. Bone Marrow Transpl.

[19] Bokhari SW, Watson L, Nagra S, Cook M, Byrne JL, Craddock C (2012). Role of HCT-comorbidity index, age and disease status at transplantation in predicting survival and non-relapse mortality in patients with myelodysplasia and leukemia undergoing reduced-intensity-conditioning hemopoietic progenitor cell transplantation. Bone Marrow Transpl.

[20] Michelis FV, Messner HA, Atenafu EG, McGillis L, Lambie A, Uhm J (2015). Patient age, remission status and HCT-CI in a combined score are prognostic for patients with AML undergoing allogeneic hematopoietic cell transplantation in CR1 and CR2. Bone Marrow Transpl.

[21] Grimm J, Jentzsch M, Bill M, Goldmann K, Schulz J, Niederwieser D (2020). Prognostic impact of the ELN2017 risk classification in patients with AML receiving allogeneic transplantation. Blood Adv.

[22] Bill M, Grimm J, Jentzsch M, Kloss L, Goldmann K, Schulz J (2018). Digital droplet PCR-based absolute quantification of pre-transplant NPM1 mutation burden predicts relapse in acute myeloid leukemia patients. Ann Hematol.

[23] Jentzsch M, Bill M, Grimm J, Schulz J, Goldmann K, Beinicke S, et al. High BAALC copy numbers in peripheral blood prior to allogeneic transplantation predict early relapse in acute myeloid leukemia patients. Oncotarget. 2017;8:87944–54. 10.18632/oncotarget.21322 PMC5675684 29152132

[24] Jentzsch M, Bill M, Grimm J, Schulz J, Beinicke S, Häntschel J (2019). Prognostic Impact of Blood MN1 Copy Numbers Before Allogeneic Stem Cell Transplantation in Patients With Acute Myeloid. Leuk Hemasphere.

[25] Lange T, Hubmann M, Burkhardt R, Franke GN, Cross M, Scholz M (2011). Monitoring of WT1 expression in PB and CD34 donor chimerism of BM predicts early relapse in AML and MDS patients after hematopoietic cell transplantation with reduced-intensity conditioning. Leukemia.

[26] Jentzsch M, Grimm J, Bill M, Brauer D, Backhaus D, Schulz J (2021). Prognostic relevance of remission and measurable residual disease status in AML patients prior to reduced intensity or non-myeloablative allogeneic stem cell transplantation. Blood Cancer J.

[27] National Marrow Donor Program and The Medical College of Wisconsin. Appendix M—The Hematopoietic Cell Transplant-Co-morbidity Index (HCT-CI). https://www.cibmtr.org/DataManagement/TrainingReference/Manuals/DataManagement/Documents/appendix-m.pdf, 2012.

[28] Sorror ML (2013). How I assess comorbidities before hematopoietic cell transplantation. Blood.

[29] R Development Core Team. R: a language and environment for statistical computing. Vienna, Austria: R Foundation for Statistical Computing (2017). http://www.R-project.org. Accessed 15 April 2022.

[30] Kaplan EL, Meier P (1958). Nonparametric Estimation from Incomplete Observations. J Am Stat Assoc.

[31] Gray RJ (1988). Class of K-Sample Tests for Comparing the Cumulative Incidence of a Competing Risk. Ann Stat.

[32] Muffly L, Pasquini MC, Martens M, Brazauskas R, Zhu X, Adekola K (2017). Increasing use of allogeneic hematopoietic cell transplantation in patients aged 70 years and older in the United States. Blood.

[33] Magliano G, Bacigalupo A (2020). Allogeneic hematopoietic stem cell transplantation for acute myeloid leukemia of the elderly: Review of literature and new perspectives. Mediterr J Hematol Infect Dis.

[34] Alyea EP, Kim HT, Ho V, Cutler C, DeAngelo DJ, Stone R (2006). Impact of Conditioning Regimen Intensity on Outcome of Allogeneic Hematopoietic Cell Transplantation for Advanced Acute Myelogenous Leukemia and Myelodysplastic Syndrome. Biol Blood Marrow Transpl.

[35] Veeraputhiran M, Yang L, Sundaram V, Arai S, Lowsky R, Miklos D (2017). Validation of the Hematopoietic Cell Transplantation–Specific Comorbidity Index in Nonmyeloablative Allogeneic Stem Cell Transplantation. Biol Blood Marrow Transpl.

[36] Versluis J, Labopin M, Niederwieser D, Socie G, Schlenk RF, Milpied N (2015). Prediction of non-relapse mortality in recipients of reduced intensity conditioning allogeneic stem cell transplantation with AML in first complete remission. Leukemia.

[37] Barba P, Piñana LJ, Martino R, Valcárcel D, Amorós A, Sureda A (2010). Comparison of Two Pretransplant Predictive Models and a Flexible HCT-CI Using Different Cut off Points to Determine Low-, Intermediate-, and High-Risk Groups: The Flexible HCT-CI Is the Best Predictor of NRM and OS in a Population of Patients Undergoing allo-RIC. Biol Blood Marrow Transpl.

[38] McClune BL, Weisdorf DJ, Pedersen TL, Tunes da Silva G, Tallman MS, Sierra J (2010). Effect of age on outcome of reduced-intensity hematopoietic cell transplantation for older patients with acute myeloid leukemia in first complete remission or with myelodysplastic syndrome. J Clin Oncol.

[39] Sorror ML, Sandmaier BM, Storer BE, Franke GN, Laport GG, Chauncey TR (2011). Long-term Outcomes Among Older Patients Following Nonmyeloablative Conditioning. JAMA.

[40] Castagna L, Fürst S, Marchetti N, El Cheikh J, Faucher C, Mohty M (2011). Retrospective analysis of common scoring systems and outcome in patients older than 60 years treated with reduced-intensity conditioning regimen and alloSCT. Bone Marrow Transpl.

[41] Xhaard A, Porcher R, Chien JW, de Latour RP, Robin M, Ribaud P (2008). Impact of comorbidity indexes on non-relapse mortality. Leukemia.

[42] Muffly LS, Kocherginsky M, Stock W, Chu Q, Bishop MR, Godley LA (2014). Geriatric assessment to predict survival in older allogeneic hematopoietic cell transplantation recipients. Haematologica.

[43] Vucinic V, Ruhnke L, Sockel K, Röhnert MA, Backhaus D, Brauer D (2021). The Diagnostic Red Blood Cell Distribution Width as a Prognostic Factor in Acute Myeloid Leukemia. Blood Adv.

[44] Brauer D, Backhaus D, Pointner R, Vucinic V, Niederwieser D, Platzbecker U (2021). Nutritional Status at Diagnosis and Pre-transplant Weight Loss Impact Outcomes of Acute Myeloid Leukemia Patients following Allogeneic Stem Cell Transplantation. Hemasphere.

[45] Ando T, Yamazaki E, Ogusa E, Ishii Y, Yamamoto W, Motohashi K (2017). Body mass index is a prognostic factor in adult patients with acute myeloid leukemia. Int J Hematol.

[46] Sandmaier BM, Kornblit B, Storer BE, Olesen G, Maris MB, Langston AA (2019). Addition of sirolimus to cyclosporine and mycophenolate mofetil-based graft-versus-host disease prophylaxis after unrelated nonmyeloablative hematopoietic cell transplantation: A multicentre phase III randomized trial. Lancet Haematol.

[47] Ziakas PD, Zervou FN, Zacharioudakis IM, Mylonakis E (2014). Graft-versus-host disease prophylaxis after transplantation: A network meta-analysis. PLoS One.

[48] Marty FM, Ljungman P, Chemaly RF, Maertens J, Dadwal SS, Duarte RF (2017). Letermovir Prophylaxis for Cytomegalovirus in Hematopoietic-Cell Transplantation. N. Eng J Med.

