# Supplementary material for: A high hematopoietic cell transplantation comorbidity Index (HCT-CI) does not impair outcomes after non-myeloablative allogeneic stem cell transplantation in acute myeloid leukemia patients 60 years or older
Source: Bone Marrow Transplant. 2022 Oct 4;58(1):30–8. doi: 10.1038/s41409-022-01833-0 (PMC9812784; doi:10.1038/s41409-022-01833-0)
Supplement: Supplementary file 1 — Supplemental material [file 41409_2022_1833_MOESM1_ESM.docx]

**SUPPLEMENTARY INFORMATION**

**“A high Hematopoietic Cell Transplantation Comorbidity Index (HCT-CI) does not impair Outcomes after Non-Myeloablative Allogeneic Stem Cell Transplantation in Acute Myeloid Leukemia Patients 60 Years or older”**

**Backhaus *et al.***

**Induction therapies**

The majority of AML patients received standard cytarabine-based induction protocols, i.e. with conventional 7+3 (n=52), conventional 7+3 with midostaurin (n=1), or sequential azacytidine and OSHO induction (n=36); were treated within or according to the OSHO studies (#0612 or #069,3 under or over 60 years, n=194), or the quantum first trial (NCT02668653, n=2). Three patients received azacitidine alone, and one patient azacitidine in combination with venetoclax.

**Conditioning Regimen, GvHD prophylaxis, GvHD criteria, and prophylaxis against infections**

Patients received a conditioning regimen consisting of fludarabine 30 mg/m^2^ day -4 to -2 and 2 or 3 Gy total body irradiation day 0 before transplantation. Graft versus host disease (GvHD) prophylaxis started day -1 with cyclosporine 2.5 mg/kg body weight twice daily, further doses twice daily were adjusted to the intended trough level of 200 ng/ml measured each day before administration of the morning dose until reaching steady state. After discharging patients from the hospital controls continued at least weekly or more intense if required (e.g. pharmacokinetically interacting drugs). Tapering was ended on day +180, if signs of GvHD were absent. Mycofenolate mofetil was administered starting day 1 with 1000 mg twice daily until day +28 followed by tapering with 500 mg each two-week interval for sibling donors and three daily doses until day +40 for matched unrelated donors.

Patients were evaluated for presence of acute GvHD and chronic GvHD using established criteria of the Glucksberg grading system. Immunosuppression was prolonged or extended with systemic steroids in cases of GvHD (grade > 2 according to Glucksberg grading system). Requirement for acute GvHD was engraftment while requirement for chronic GvHD was engraftment and survival for at least 100 days after HSCT. Anti-infective prophylaxis was started parallel to the conditioning regimen with valaciclovir 500 mg orally twice daily, cotrimoxazole twice weekly 960 mg twice daily after day +30 and hematologic regeneration, ciprofloxacine 500 mg twice daily, and fluconazole 400 mg daily until cessation of immunosuppressive drugs. ﻿Cytomegalovirus (CMV) infection was screened with quantitative polymerase chain reaction (PCR) twice weekly and after dischargement from hospital weekly in the outpatient department. None of the patients undergoing NMA-HSCT received *in vivo* T-cell depletion.

**Regeneration after HSCT**

**﻿**Leukocyte and platelet engraftment were defined as white blood count (WBC) >1.0 x 10^9^ per liter and on two consecutive days and platelets >50 x10^9^ per liter without transfusion. 27 and 41 patients had no nadir below the here defined parameters and were counted as regeneration at day 0. Eight patients did not engraft and were excluded. Median days and range of included patients are shown in Table 1 in each HCT-CI risk group.

**Definition of remission endpoints and secondary AML**

Complete remission (CR) was defined as the presence of <5% of blasts in bone marrow (BM), neutrophils >1.0 x 10^9^/L, platelets >100 x 10^9^/L, absence of blasts with Auer rods, independence of blood transfusion and no extramedullary disease. CR with incomplete peripheral recovery (CRi) was defined as CR with platelets <100 x 10^9^/L or neutrophils <1.0 x 10^9^/L. The presence of CR or CRi was confirmed within 28 days prior to HSCT by bone marrow and peripheral blood analysis.

Secondary AML was defined as AML developing after an antecedent myeloid neoplasm (i.e., myelodysplastic syndrome, myeloproliferative neoplasm, or MDS/MPN) or developing after exposure to chemotherapy or radiation applied for the treatment of lymphomas, solid tumors, or autoimmune diseases.

**Further information on prevalence of malignancies**

Additional overview on prevalence of malignancies in female and male patients in the whole cohort are shown in Supplementary Figure S2.

**Supplementary Tables**

**Supplementary Table S1. Prevalence of tumors in the patient cohort (A) female and (B) male patients**

**A**

|  | female | | | | |
| --- | --- | --- | --- | --- | --- |
| tumor entity | n | radiation (n) | chemotherapy (n) (n) | only surgery (n) |  |
| breast cancer | 21 | 15 | 12 | 1 |  |
| endometrial carcinoma | 5 | 4 | 1 | 1 |  |
| cervix carcinoma | 6 | 1 | 0 | 4 |  |
| ovarian carcinoma | 3 | 1 | 2 | 0 |  |
| colorectal carcinoma | 2 | 0 | 2 | 0 |  |
| gastric carcinoma | 1 | 0 | 1 | 0 |  |
| bronchial-carcinoma | 1 | 1 | 1 | 0 |  |
| thyroid carcinoma | 1 | 1 | 0 | 0 |  |
| malignant melanoma | 1 | 0 | 0 | 1 |  |
| lymphoma | 5 | 0 | 5 | 0 |  |
| other | 3 | 2 | 3 | 0 |  |

**B**

|  | male | | | |
| --- | --- | --- | --- | --- |
| tumor entity | n | radiation (n) | chemotherapy (n) | only surgery (n) |
| prostate carcinoma | 4 | 1 | 0 | 3 |
| esophageal carcinoma | 1 | 1 | 1 | 0 |
| colorectal carcinoma | 5 | 2 | 3 | 2 |
| gastric carcinoma | 2 | 1 | 1 | 2 |
| urothelial carcinoma | 5 | 0 | 1 | 4 |
| malignant melanoma | 1 | 0 | 1 | 0 |
| lymphoma | 3 | 0 | 3 | 0 |
| seminoma/non-seminoma | 2 | 0 | 2 | 0 |
| other* | 3 | 2 | 2 | 1 |

*other: cancer of unknown primary, musculoskeletal cancers, and teratoma.

**Supplementary Table S2. Relapse and NRM including GvHD in the three HCT-CI risk groups.**

|  | HCT-CI  0 points | HCT-CI  1/2 points | HCT-CI  ≥ 3 points | *P*  (0 *vs* 1/2) | *P*  (1/2 *vs* 3) | *P*  (0 *vs* 3) |
| --- | --- | --- | --- | --- | --- | --- |
| acute GvHD ≥ grade 2, n (%) | | | | | | |
| absent  present | 52 (55.3)  42 (44.7) | 55 (74.3)  19 (25.7) | 53 (63.9)  30 (36.1) | .02 | .17 | .28 |
| chronic GvHD, n (%) | | | | | | |
| absent  limited  extensive | 23 (31.5)  12 (16.4)  38 (52.1) | 23 (39.0)  5 (8.5)  31 (52.5) | 15 (24.2)  15 (24.2)  32 51.6) | .34 | .04 | .45 |
| causes of death, n (%) | | | | | | |
| relapse  GvHD  infection  other | 31 (53.4)  10 (17.2)  13 (22.4)  4 (7.0) | 29 (59.2)  10 (20.4)  5 (10.2)  5 (10.2) | 27 (55.1)  13 (26.5)  7 (14.3)  2 (4.1) | .39 | .56 | .49 |
| Abbreviations: GvHD, Graft versus host disease; HCT-CI, Hematopoietic stem cell comorbidity index | | | | | | |

**Supplementary Table S3. Outcome *P* values for the HCT-CI risk score as continuous variable**

|  | NRM, *P* | OS, *P* |  |  |  |  |  |
| --- | --- | --- | --- | --- | --- | --- | --- |
| All patients | 0.63 | 0.29 |  |  |  |  | |
| MRD |  |  |  |  |  |  | |
| positive  negative | 0.83  0.53 | 0.15  0.63 |  |  |  |  | |
| ELN2017 genetic group |  |  |  |  |  |  | |
| favorable  intermediate  adverse | 0.88  0.89  0.87 | 0.17  0.50  0.89 |  |  |  |  | |
| Disease origin |  |  |  |  |  |  | |
| *De novo* AML  Secondary AML | 0.64  0.20 | 0.73  0.22 |  |  |  |  | |
| Abbreviations: AML, Acute myeloid leukemia; ELN2017, European leukemia net classification 2017; MRD, Measurable residual disease | | | | | | | |

**Supplementary Figures.**

**Supplementary Figure S1**

**Supplementary Figure S1:** Cumulative incidence of relapse and non-relapse mortality according to timepoint of allogeneic HSCT (prior to vs after median date of HSCT in the analyzed cohort, November 15, 2011).

**Supplementary Figure S2**

**A
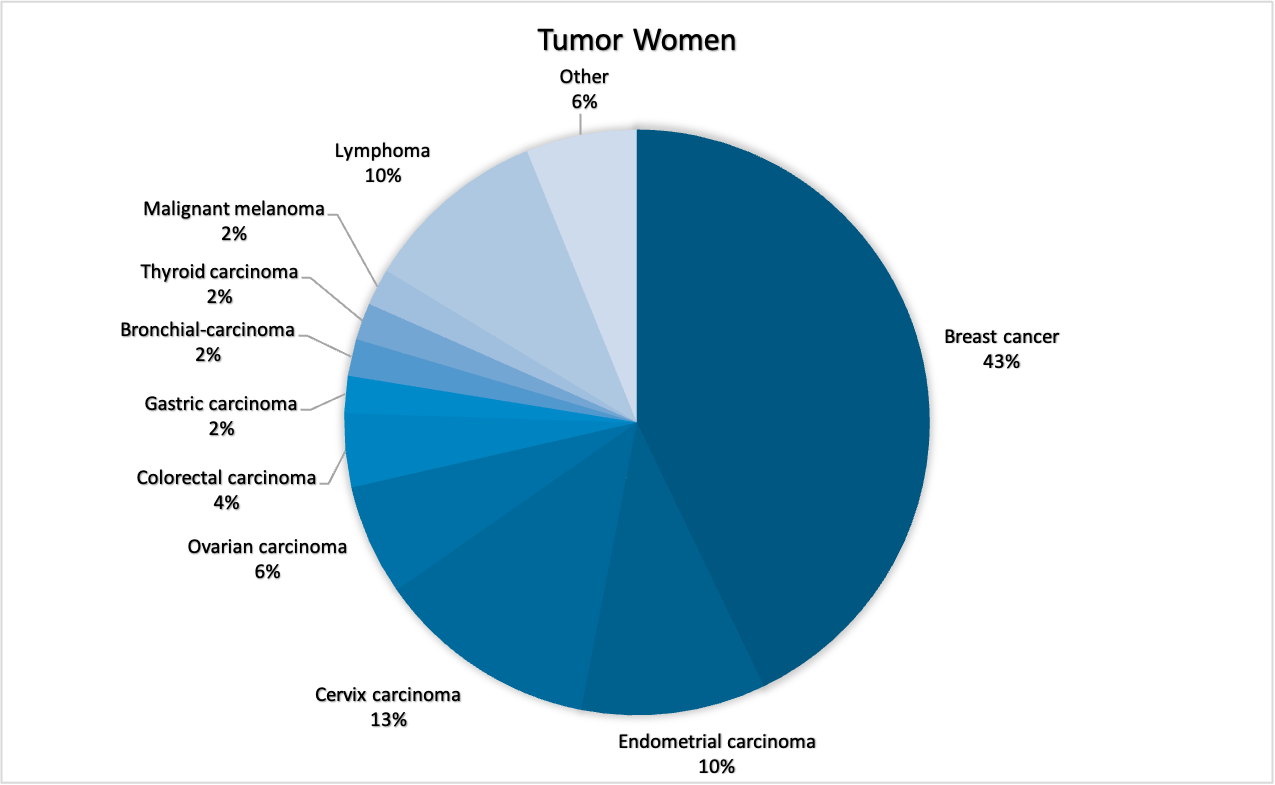
**

**B**


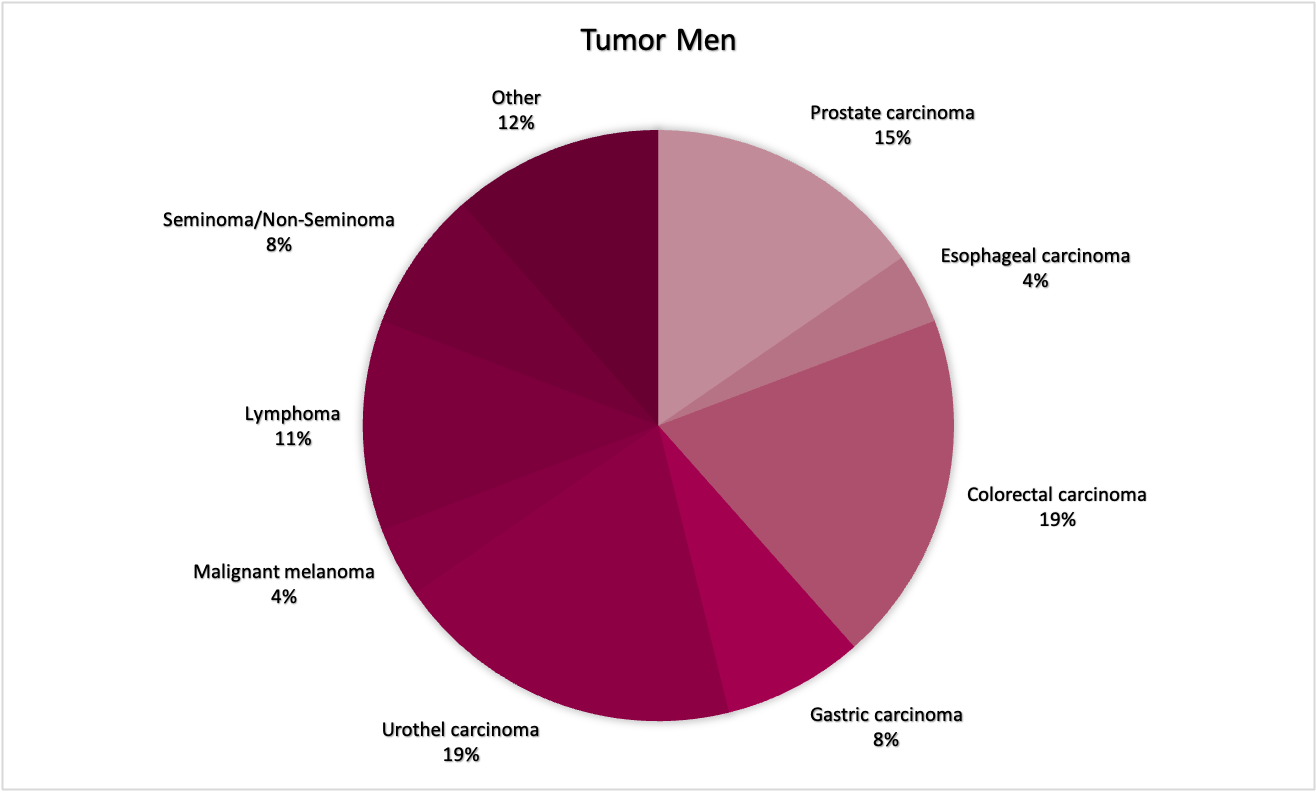


**Supplementary Figure S2.** Prevalence of prior malignancies in (A) female and (B) male patients.

**Supplementary Figure S3.**

**Supplementary Figure S3.** Overall survival at 5 years for AML patients receiving NMA-HSCT for **(A)** the whole patient cohort and **(B)** MRD-negative and MRD-positive patients at HSCT.

**Supplementary Figure S4.**

**Supplementary Figure S4.** Receiver Operator characteristics (ROC) curves for the prediction of **(A)** Non-relapse mortality and **(B)** Overall survival by the HCT-CI risk score as continuous variable.

**Supplementary Figure S5**

**Supplementary Figure S5.** Non-Relapse Mortality, and Overall Survival after NMA-HSCT according to the three HCT-CI risk groups in **(A, B)** patients with secondary AML, and **(C, D)** patients with *de novo* AML.

**Supplementary Figure S6**

**Supplementary Figure S6.** Receiver Operator characteristics (ROC) curves for the prediction of non-relapse mortality and Overall survival by the HCT-CI score as continuous variable in **(A, B)** MRD-negative and **(C, D)** MRD-positive patients.

**Supplementary Figure S7**

**
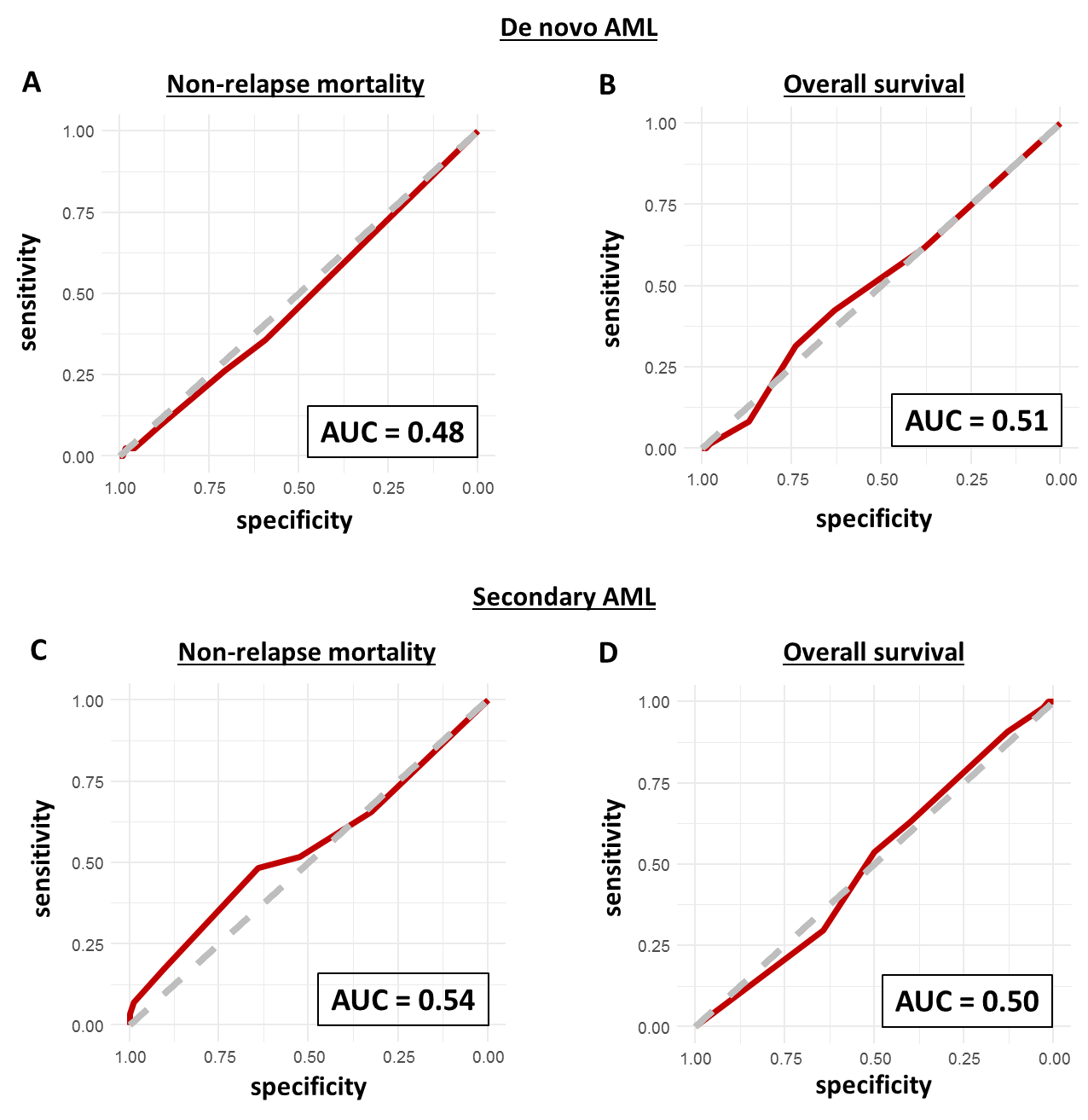
**

**Supplementary Figure S7.** Receiver Operator characteristics (ROC) curves for the prediction of non-relapse mortality and overall survival by the HCT-CI score as continuous variable in **(A, B)** *de novo* AML and **(C, D)** secondary AML.

**Supplementary Figure S8**

**Supplementary Figure S8.** Receiver Operator characteristics (ROC) curves for the prediction of non-relapse mortality and overall survival by the HCT-CI score as continuous variable in **(A, B)** ELN2017 favorable **(C, D)** ELN2017 intermediate, and (E, F) ELN2017 adverse risk patients.

**Supplementary Figure S9**

**Supplementary Figure S9.** Overall Survival after NMA-HSCT according to **(A)** the RDW at diagnosis (high *vs* low, 20.7 cut), and **(B)** weight loss between diagnosis and HSCT (≥ 2 *vs* < 2 BMI points)
